# Supplementary material for: Real-World Outcomes and Biomarker Analysis Based on Routine Clinical, Laboratory, and Pathologic Parameters in Metastatic or Unresectable Esophageal Cancer Treated with First-Line Anti-PD-1 Plus Fluoropyrimidine and Platinum
Source: Cancers (Basel). 2025 Sep 28;17(19):3149. doi: 10.3390/cancers17193149 (PMC12524331; doi:10.3390/cancers17193149)
Supplement: Supplementary file 1 [file cancers-17-03149-s001.zip › Supplementary_material_250926.pdf]

Supplementary Materials

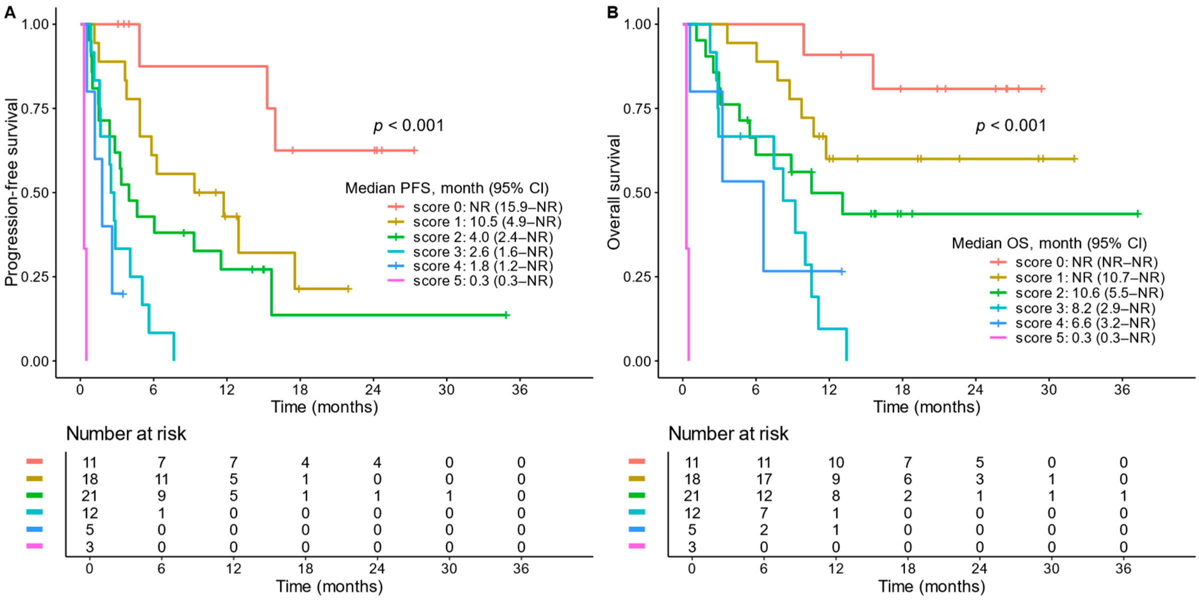

Figure S1. Kaplan–Meier curves according to total risk score (range, 0–5).

(**A**) Progression-free survival (PFS).

(**B**) Overall survival (OS).

p values were calculated using the log-rank test. Tick marks indicate censored observations. CI, confidence interval; NR, not reached.

Table S1. Duration of treatment and dose modifications

Treatment	Median Treatment duration, months (range)	Dose Reduction, n (%)	Dose Delays*, n (%)
Pembrolizumab plus chemotherapy (n=58)	5.1 (0.7-29.6)		
Pembrolizumab	5.1 (0.7-29.6)	0 (0.0%)	29 (50.0%)
Cisplatin	4.3 (0.7-9.9)	46 (79.3%)	27 (46.6%)
5-FU or capecitabine	4.8 (0.8-29.7)	41 (70.7%)	28 (48.3%)
Nivolumab plus chemotherapy (n=29)	4.7 (0.9-18.2)		
Nivolumab	4.7 (0.9-18.2)	2 (6.9%)	12 (41.4%)
Cisplatin	4.6 (0.0-14.9)	18 (62.1%)	13 (44.8%)
5-FU or capecitabine	4.7 (1.0-18.3)	19 (65.5%)	13 (44.8%)

* A dose delay was defined as initiation of the subsequent treatment cycle more than 3 days after the scheduled date.

5-FU, 5-fluorouracil.

Table S2. Post-discontinuation anticancer therapy

**Therapy**	**No. (%) (n = 87)**			
Any subsequent therapy	37 (42.5)			
Definitive chemoradiotherapy	1 (1.1)			
Systemic therapy	**Total**	**2nd line**	**3rd line**	**4th line**
Immunotherapy				
Nivolumab	2 (2.3)	0 (0.0)	1 (1.1)	1 (1.1)
Anti-4-1BB/anti-PD-L1 bi-specific mAb	2 (2.3)	0 (0.0)	2 (2.3)	0 (0.0)
Tislelizumab	1 (1.1)	1 (1.1)	0 (0.0)	0 (0.0)
Cytotoxic chemotherapy				
Docetaxel	33 (37.9)	32 (36.8)	1 (1.1)	0 (0.0)
Irinotecan + cisplatin	11 (12.6)	1 (1.1)	10 (11.5)	0 (0.0)
Paclitaxel	1 (1.1)	1 (1.1)	0 (0.0)	0 (0.0)
Docetaxel + cisplatin	1 (1.1)	1 (1.1)	0 (0.0)	0 (0.0)
Vinorelbine	1 (1.1)	0 (0.0)	1 (1.1)	0 (0.0)
Vinorelbine + cisplatin	1 (1.1)	0 (0.0)	1 (1.1)	0 (0.0)
Epirubicin + mitomycin C + 5-FU	1 (1.1)	0 (0.0)	0 (0.0)	1 (1.1)

mAb, monoclonal antibody; 5-FU, 5-fluorouracil

Table S3. Treatment response and survival outcomes

	**Measurable disease (n=82)**	**Non-measurable disease (n=5)**
Best overall response		
CR	2 (2.4%)	2 (40%)
PR	38 (46.3%)	–
SD	22 (26.8%)	–
Non-CR/non-PD	–	3 (60%)
PD	15 (18.3%)	0 (0%)
NE*	5 (6.1%)	0 (0%)
Objective response rate, n (%) (95% CI)	42 (48.3%) (37.4-59.2)	
Disease control rate, n (%) (95% CI)	67 (77.0%) (66.8-85.3)	
Median duration of response (95% CI)	11.8 months (6.9-not reached)	
Median progression-free survival (95% CI)	5.6 months (4.5–8.7)	
Median overall survival (95% CI)	13.1 months (10.6–not reached)	
6-month progression-free survival (95% CI)	46.7% (36.9–58.9)	
1-year progression-free survival (95% CI)	29.0% (20.4–41.2).	
6-month overall survival (95% CI)	78.7% (70.5–88.0)	
1-year overall survival (95% CI)	52.1% (42.0–64.6)	

* Not evaluable: defined as absence of follow-up imaging to assess response.
CR, complete response; PR, partial response; SD, stable disease; PD, progressive disease; CI, confidence interval.

Real-World Data Source, Variable Dictionary, and Analysis Overview

1. Study Registration

This study was not registered in a public clinical trial registry, as it was a retrospective, observational study based on clinical data extracted from institutional electronic medical records. Patient-level data were de-identified prior to analysis in accordance with institutional policies and IRB approvals.

2. Data Source and Ownership

Clinical data were collected independently from the electronic medical record systems of the following institutions:

- Asan Medical Center, Seoul, Republic of Korea
- Severance Hospital, Yonsei University Health System, Seoul, Republic of Korea

Each institution maintains full ownership and governance of its respective dataset. No inter-institutional data linkage was performed, and all data were deidentified prior to transfer and analysis.

3. Data Collection and Management

Data were extracted using predefined templates and included demographic, clinical, laboratory, and pathologic variables. All data were curated locally at each institution under the oversight of the site principal investigator. Quality control procedures included:

- Double-checking of key clinical dates (e.g., treatment initiation, response assessment)
- Logical consistency checks for laboratory and pathologic variables
- Missing data review and documentation

The final database was locked for analysis on April 4, 2025.

4. Variable Dictionary and Derivation Methods

A comprehensive data dictionary, including variable names, definitions, coding schemes, and derivation methods, is provided below:

Variable Name	Definition	Source	Derived/Coded	Method of Derivation or Coding	Validation Method
Age	Age at initiation of ICI	EMR	Raw	Extracted from birthdate and treatment start date	Cross-checked by site PIs
Sex	Biological sex	EMR	Raw	Directly extracted	Not applicable
Regimen	First-line palliative systemic therapy regimen (e.g., pembrolizumab/FP, nivolumab/FP, pembrolizumab/XP, nivolumab/XP)	EMR	Raw	Extracted from medication administration records	Cross-checked by site PIs
ECOG PS	ECOG performance status	EMR (clinician documentation)	Raw	As recorded by treating physician	Cross-checked by site PIs
Smoking history	History of cigarette smoking status (never, former, current)	EMR (clinician documentation)	Coded	As recorded by treating physician	Cross-checked by site PIs
Histology type	Esophageal cancer	Pathology report	Raw	As reported in initial	Pathologist-

Variable Name	Definition	Source	Derived/Coded	Method of Derivation or Coding	Validation Method
	histologic type (squamous vs non-squamous)			pathology	confirmed
Tumor differentiation	Histologic differentiation of esophageal cancer (well, moderately, poorly, undifferentiated, unknown)	Pathology report	Raw	As reported in initial pathology	Pathologist-confirmed
Disease status	Metastatic vs. locally advanced unresectable	EMR, imaging report	Coded	Categorized based on initial staging	Cross-checked by site PIs
Metastatic sites	Type and location of metastatic organs at baseline	Imaging review	Coded	Categorized per investigator assessment	Cross-checked by site PIs
PD-L1 CPS	Combined Positive Score	Pathology report	Raw	As reported from IHC test	Pathologist-confirmed
PD-L1 TPS	Tumor Proportion Score (%)	Pathology report	Raw	As reported from IHC test	Pathologist-confirmed
TMB	Tumor Mutation Burden (mut/Mb)	NGS report	Raw	OncoPanel or TSO-500	Validated by molecular pathology lab
MSI status	Microsatellite instability	Pathology report, NGS report, Molecular pathology report	Raw	Determined using next-generation sequencing (NGS), immunohistochemistry (IHC), or polymerase chain reaction (PCR)	Confirmed by a pathologist (IHC) or validated by a molecular pathology lab (PCR/NGS)
Prior therapies	Pre-ICI local therapy for esophageal cancer (e.g., CCRT, RT, chemotherapy)	EMR	Raw	Based on medication records and physician documentation	Cross-checked by site PIs
Hb	Hemoglobin (g/dL)	EMR	Raw	Latest value prior to ICI initiation	Manually extracted and verified
CRP	C-reactive protein (mg/L)	EMR	Raw	Latest value prior to ICI initiation	Manually extracted and verified
NLR	Neutrophil-to-lymphocyte ratio	Derived from lab values	Derived	Absolute neutrophil count / lymphocyte count	Formula-based; reviewed in dataset
PLR	Platelet-to-lymphocyte ratio	Derived from lab values	Derived	Absolute platelet count / lymphocyte count	Formula-based; reviewed in dataset
Na	Sodium (mmol/L)	EMR	Raw	Latest value prior to ICI initiation	Manually extracted and verified
Albumin	Albumin (g/dL)	EMR	Raw	Latest value prior to ICI initiation	Manually extracted and verified
IO treatment duration	Median duration of pembrolizumab or nivolumab treatment	EMR	Derived	Calculated based on administration dates	Reviewed in dataset
IO dose reduction	Dose reduction of	EMR	Derived	Calculated from dosing	Reviewed in

Variable Name	Definition	Source	Derived/Coded	Method of Derivation or Coding	Validation Method
	pembrolizumab or nivolumab			records	dataset
IO dose delay	Dose delay of pembrolizumab or nivolumab	EMR	Derived	Calculated from scheduled vs actual administration dates	Reviewed in dataset
Cisplatin duration	Median duration of cisplatin treatment	EMR	Derived	Calculated based on administration dates	Reviewed in dataset
Cisplatin dose reduction	Dose reduction of cisplatin	EMR	Derived	Calculated from dosing records	Reviewed in dataset
Cisplatin dose delay	Dose delay of cisplatin	EMR	Derived	Calculated from scheduled vs actual administration dates	Reviewed in dataset
5-FU/Capecitabine duration	Median duration of 5-fluorouracil or capecitabine treatment	EMR	Derived	Calculated based on administration dates	Reviewed in dataset
5-FU/Capecitabine dose reduction	Dose reduction of 5-fluorouracil or capecitabine	EMR	Derived	Calculated from dosing records	Reviewed in dataset
5-FU/Capecitabine dose delay	Dose delay of 5-fluorouracil or capecitabine	EMR	Derived	Calculated from scheduled vs actual administration dates	Reviewed in dataset
Reason for regimen discontinuation	Reason for stopping 1st-line regimen (e.g., ongoing, PD, toxicity, follow-up loss, patient will, others)	EMR	Coded	Classified from EMR documentation	Cross-checked by site PIs
Measurable lesion	Presence of measurable lesion per RECIST 1.1	imaging review	Coded	Assessed based on RECIST criteria	Cross-checked by site PIs
ORR	Objective Response Rate (RECIST v1.1)	Imaging review	Coded	CR or PR per investigator assessment	Double-reviewed by site PIs
DCR	Disease Control Rate (RECIST v1.1)	Imaging review	Coded	CR or PR or SD per investigator assessment	Double-reviewed by site PIs
DoR	Duration of response (RECIST v1.1)	imaging review	Coded	the time from first CR or PR to progression or death	Double-reviewed by site PIs
PFS	Progression-free survival (months)	EMR	Derived	Time from ICI start to disease progression, death, or last follow-up	Censoring reviewed by site PIs
OS	Overall survival (months)	EMR	Derived	Time from ICI start to death or last follow-up	Censoring reviewed by site PIs
Further treatment	Subsequent therapy after 1st-line ICI	EMR	Raw	Based on medication records and physician documentation	Cross-checked by site PIs
Antibiotic exposure pre-IO	Days of antibiotic use within 30 days prior to ICI initiation	EMR	Raw	Based on medication records and physician documentation	Cross-checked by site PIs
Adverse events	Collected AEs including type, maximum grade,	EMR	Coded	Annotated by CTCAE criteria	Cross-checked by site PIs

Variable Name	Definition	Source	Derived/Coded	Method of Derivation or Coding	Validation Method
	SAE, and irAE status				

5. Data Accessibility
Access to raw or patient-level data is restricted in accordance with institutional and IRB policies. However, deidentified datasets and metadata may be made available upon reasonable request to the corresponding author. Data sharing is subject to institutional approval and execution of a data use agreement.

6. Timing of Core Variables
Time points for all core variables were standardized relative to treatment initiation. Baseline clinical and laboratory variables were assessed within 7 days prior to first-line ICI initiation. Biomarkers (PD-L1, TMB, MSI) were assessed from pretreatment tumor tissue. Response and survival outcomes were measured during and after treatment based on clinical follow-up.

7. Risk of Bias and Mitigation Strategies
To reduce selection bias, consecutive eligible patients were included. Predefined templates were used for data extraction, and key variables were cross-validated by site investigators to minimize information bias. Prognostic modeling included multivariable adjustment for clinical confounders, and multicollinearity was avoided by excluding redundant variables.

8. Biomarker Assessment
PD-L1 expression was assessed using the 22C3 or 28-8 pharmDx kits (Agilent) with CPS and TPS scoring. TMB was measured via NGS using the OncoPanel (Asan Medical Center) or TSO-500 (Severance Hospital). All assays were conducted in certified institutional labs and interpreted by board-certified pathologists or molecular diagnostics teams. Biomarker testing was performed on pretreatment archival tissue samples.

9. Prespecified vs. Post hoc Analyses
Prespecified analyses included the evaluation of primary clinical endpoints (objective response rate, disease control rate, progression-free survival, and overall survival) and the assessment of predefined prognostic factors such as ECOG performance status, baseline CRP level, and PD-L1 CPS stratification (<10, 10–49, and ≥50).
Post hoc analyses included the selection of optimal cut-off values for continuous laboratory parameters using the maximally selected log-rank statistic, and the development of a simplified prognostic scoring model based on variables found to be independently associated with survival outcomes in multivariable analysis. These analyses were exploratory in nature and not defined in the initial study plan.

10. Internal and External Validity, and Sensitivity Analyses
Internal validity was supported by the inclusion of all eligible patients treated with a defined

period, and potential confounding was addressed using multivariable Cox regression. Although no formal sensitivity analyses were performed, the overall consistency of the findings supports the robustness of the results.

External validation of the prognostic scoring model was not feasible due to the unavailability of independent datasets with comparable variables and treatment regimens. Nevertheless, the inclusion of real-world patients—such as those with poor performance status or multiple comorbidities, who are often underrepresented in clinical trials—may enhance the generalizability of our findings.
